# The ‘Compleat Physician’ and Experimentation in Medicines: Everard Maynwaring (*c*.1629–1713) and the Restoration Debate on Medical Practice in London

**DOI:** 10.1017/mdh.2018.2

**Published:** 2018-04

**Authors:** Jonathan Barry

**Affiliations:** Department of History, Amory Building, University of Exeter, EX4 4RJ, UK

**Keywords:** Restoration, Physicians, Chemical medicines, Experimental medicines, Galenism, Universal medicines

## Abstract

Restoration London saw a wave of publications by physicians advocating that the ‘compleat physician’ should be one who experimented and produced his own medicines. Only thus, they argued, could the medical hierarchy be restored and medical authority re-established on a defensible basis. This article seeks to explain the context for this unusual approach, and why it failed to attract mainstream physicians by the end of the century, by considering the sixty-year career of one of its leading advocates, Everard Maynwaring (*c*.1629–1713), a prolific medical author, and what his own failure to enter the medical establishment may show about the problems inherent in this model for the physician. A university-trained gentleman physician who converted to chymical medicine *c*.1660, Maynwaring published learned and relatively unpolemical texts to persuade both medical and lay audiences of the superiority of experimental medicine as a mode of learned practice, yet could not easily reconcile this with the advocacy and sale of his own chymical medicines (especially as he focused increasingly on a small group of ‘universal medicines’) without being branded an ‘empirick’. Fragmentary evidence regarding his career suggests he became increasingly marginalised, and as an old man was reduced to advertising his cures like the ‘empiricks’ from whom he had sought to distance both himself and physicians in general.

Restoration London saw a wave of publications by physicians advocating that physicians should experiment and produce their own medicines. Only thus, they argued, could the medical hierarchy be restored and medical authority re-established on a defensible basis.^1^ This article considers the sixty-year career of one of the leading advocates of this position, Everard Maynwaring (*c*.1629–1713).^2^ In the absence of any personal papers or business materials, we are largely dependent on Maynwaring’s own writings to chart his motivations and career. He is known as a supporter of the Society of Chymical Physicians in 1665 and he was one of the more prolific medical authors (whose numerous titles mostly went through several editions). But his career and writings have never been studied, though his ideas on the history of medicine, the treatment of pain, the passions and contagion have recently been noticed.^3^ We shall see how he pioneered the combination of a learned defence of the new model of the ‘compleat physician’ with an entrepreneurial attempt to take advantage of the thriving London market for proprietary medicines, especially those based on the new ‘chymical physick’. This article also analyses what his failure to enter the medical establishment, and increasingly marginalised status, reveals about the problems inherent in this model for the physician.

Significantly, his current ODNB biography contains nothing on him after 1698, since he ceased to publish new works after the 1698 edition of his *Ignota Febris*. In this he reproduced his Cambridge MB (1 July 1652), and his Trinity College Dublin MD of 1655, because ‘having often found ill usage, and Detractions by some Men of the *Physick-Trade;* [he] hath therefore caused the following *Testimonials* to be made Publick; to stifle, and null the *Defamations,* and *Lies* that have been spread abroad, to his Prejudice, and *Loss* to many others; that else might have received the Benefit of his great Labour; beyond what the *Shops* do afford’.^4^ Maynwaring blamed this ‘ill usage’ on the fact that for thirty years he had been producing his own medicines. He must be the ‘Dr Mannwaring’ whose medicine the London College of Physicians refused to approve on 12 May 1701 because he would not reveal its composition.^5^ This exposed him to the charge of being an ‘empirick’ trading in a secret medicine, and from the 1680s the College had been objecting to physicians developing and selling their own medicines. Two different Fleet Street booksellers in 1702 advertised his book on pains (with the altered title *The latent dangerous progress and fatal events of internal pains* by ‘Everard Maynwaringe Med. D’),^6^ but I can find no further references to him for ten years until the *Spectator* of 9 July 1712 carries an advertisement for ‘Dr Maynwaringe’ who ‘undertakes the curing of such desperate and most difficult diseases wherein some very able and long-experienced physicians and surgeons in hospitals and great cities have failed in the attempt’ or refused ‘such business, wherein they have no hope of performance with credit’. Giving a list of such conditions, he promises that such cases ‘seemingly incurable to others, he receives into his care for cure and is successful by means extraordinary. At the Moor’s Head (for distinction) in Baldwin’s Garden by Gray’s Inn.’ He had been reduced in his old age to advertising his services in a style little different from the ‘empiricks’ from whom he constantly sought to differentiate himself. Finally, ‘Everard Mannering’, ‘out of Baldwin’s Gardens’, was buried at St Andrew’s, Holborn, 21 February 1712/13.^7^ Maynwaring’s sixty-year career thus neatly spans the rise and fall of this particular model of the ‘compleat physician’.

Whatever his later difficulties, Maynwaring came from an establishment background equal (if not superior) to that of most College physicians. The Maynwarings of Cheshire were an extensive gentry kinship. His grandfather, Henry Maynwaring, esquire, of Kermincham in Cheshire (d.1617), had married Elizabeth Digby (d.1624) from Stoke Dry in Rutland – probably the granddaughter of the Kenelm Digby who sat six times as Tudor MP for Rutland – and through this connection Everard got both his name and his distant cousinage of Sir Kenelm Digby, the prominent royalist and chymical experimenter, whose father Everard Digby (grandson of the MP) had been executed for his role in the Gunpowder Plot. Eleanor Maynwaring, the first wife of Elias Ashmole, another royalist chymist, was Everard’s great-uncle’s daughter.^8^ Our Everard’s father, also Kenelm (*c*.1598–1661), became rector of Gravesend in Kent, but was sequestered in 1643 as a royalist, only being restored in 1660.^9^ We have no clear evidence of Maynwaring’s own religious or political opinions (not least because he never became embroiled in any personal controversy) although his writings suggest he was a conformist to both religious and political authority, willing to serve his country medically under Cromwell, Charles II, James II and William.

Everard was educated at Gravesend Grammar School before matriculating as a sizar, aged 17, at St John’s Cambridge on 21 June 1645.^10^ He graduated MB seven years later, and then moved north close to his relatives to begin medical practice. He records in 1679 that in 1652 ‘being then for a while at Norton upon the edge of Darby-shire ... I being but a Tyro in this Art, it being in the first year of my Practice, and newly graduated Batchellor in Physick’, he had cured a patient near Sheffield.^11^ His father held the living at Norton from 1643 until early 1657, when he was forced to resign for his ‘scandalous’ royalism, though he appealed to Cromwell that he had been falsely accused.^12^ Everard then records a case as ‘a young practiser’ in ‘Maxfilde’ (Macclesfield, about 6 miles from Kermincham) in 1653 ‘where I happened to stay in that town for some time’, and a woman coming to him at Chester ‘where I then practised’ in 1658.^13^ However, between 1653 and 1657 he travelled both to America and to Ireland. In September 1664 the Dublin MD Christopher Lawrence testified to their friendship ‘abhinc diu in *America* contractae, postea hîc feliciter continuatae’.^14^ The papers of the Chester herald, Randall Holme, include a genealogy for an Everard Maynwaring ‘Dr of Physick in Ireland in 1654’.^15^ Maynwaring himself explains his MD (awarded on 5 September 1655, after public exercises on 17 August) thus: ‘Some Years after [his 1652 MB], the *Author* travelling into *Ireland*; and being in *Dublin,* at the Time of a Publick Commencement: upon producing this *Diploma* from *Cambridge;* and performing such Exercises, as the Statutes of the *University* required: He proceeded *Doctor.*’ He was probably in Ireland to serve the army as in 1689 he refers to ‘having lived in Ireland many years since; where observing the endemic diseases of that Country, which commonly begins Lienterie, and makes its transition into a Dysentery or Bloody Flux; the disease proving fatal to many; especially to those that have not been accustomed to that air and food; which alternation caused a languishing sickness and the death of many in Cromwel’s army’.^16^ He must have returned to Chester some time before January 1657.^17^

But at some date between 1658 and 1662 he moved away from the area where he had such strong kinship support and established himself in London. This coincided with a drastic change in his medical practice. It was clearly around 1660–2 that Everard renounced Galenic physic in favour of chymical medicines. In 1698 he refers to Galenic remedies for fever: ‘these I did use in the beginning of my Practice, when I was a *Prescriber,* (Forty Years ago) and *guessed* at Medicines, as others now do, that *Prescribe* to the *Shops*’.^18^ He offers a fuller account in the preface to his *Useful Discoveries and Practical Observations in Some Late Remarkable Cures of the Scurvy* (1668), discussing three medicines he has prepared against scurvy which ‘I have been reforming and improving almost seven years’, that is, since about 1661.^19^

I did eagerly apply myself to Medicine according to the ancient custom and general practice of the most learned and famous Physicians in all places (who were industrious Artists, diligent in preparing their own Medicines, until this later age) with as much curiosity as my knowledg could possibly direct. At first I was desirous to make Experiments, and be fully informed in *Galenic* Medicines, being grounded upon those Principles by my *Academic Education*, and was tenacious enough of that Doctrine, until a clearer prospect of truth did appear, gained by Observations in practical Philosophy, a serious ratiocination and strict examination of Principles and received Opinions: but being removed off that Basis, and confirmed by Chymical Tryals relating to Medicine; I deserted the *Galenic* Medicines as inferiour to what I discovered and was presented to my view, and ever since have labored in Chemical Pharmacy, as being the most excellent way of preparing Medicines.

Maynwaring then decided to set himself up in London, where chymical medicine was establishing itself as a dynamic challenger to Galenic practice. Before analysing his career there, it is necessary to sketch both the broader intellectual context of this challenge, and the specific medical politics of London at this period. Chymical medicine, associated particularly with Paracelsus and Van Helmont, was a broad and highly complex movement.^20^ Drawing sometimes on claims to an ancient learning (hermetic and/or Biblical) rivalling that of classical medicine, sometimes on a radical rejection of book learning in favour of practical experience and especially ‘labour in the fire’, this approach looked to the advancing technologies of miners, distillers and alchemists to produce new medicines to replace traditional remedies. In many cases, this went with a rejection of the traditional model of humoral imbalance as the source of disease, instead identifying diseases as external poisons attacking the vital principles of the whole body and potentially treatable either by specific remedies for specific diseases or by new universal medicines capable of strengthening the whole body to fight off invaders. To traditional Galenists these approaches seemed totally unscientific, associated with the ‘empirical’ medicines of ‘quacks’ who also offered either specific remedies for single diseases or universal panaceas. Yet, despite bitter controversies, chymical physicians and, in particular, chymical medicines gradually entered mainstream medical practice and education, notably in Montpellier and the Dutch and German-speaking universities, reflecting a growing eclecticism and empiricism in medical teaching and practice.

Two aspects of chymical medicine are critical here, when considering Maynwaring’s choice and its implications. Firstly, it arose from the traditions of ‘secrets’, particularly in alchemy. Such knowledge was only won through personal dedication to uncovering (perhaps with divine assistance through purification, but also from the inherent danger and toil of the laboratory) the secrets of nature which neither could nor should be made publicly available, even if the medicines that ensued should be shared for the common good. The inventor deserved to reap the fruits of his secrets: in the absence of a patent system and operating outside orthodox medical hierarchies, such people built careers by possessing such secrets, frequently by offering their services to courts/aristocrats (ironically, given that the movement began with Paracelsus as a socially and religious radical preference for the knowledge and needs of the common people).^21^

Secondly, chymical medicine focused on the production of *medicines*, which both stimulated, but also benefited from, the expansion of a commercial market for medicines, especially medicines which were not made on demand in an apothecary’s shop to meet the specific prescription of a specific physician for a specific patient in a specific illness, but were pre-produced and even commercially packaged and sold as branded items.^22^ A specific trade of ‘chymists’ emerged to produce such medicines. Both their specific and universal remedies were necessarily better suited to commercial marketing than were the Galenical remedies, as they did not require tailoring for an individual constitution. Evidence for growing expenditure on medical services during the seventeenth century, while hard to interpret definitively, suggests a growing market penetration (both socially and geographically) associated with more people having access to, and spending money on, medicines, and (more tentatively) a particular growth in apothecary numbers (though also of surgeons in maritime or military settings).^23^

As Cook and Wear have both identified, this growth of public demand for ‘medicines’ for specific diseases (including the demand from military leaders) triggered a crisis in Galenic medicine which had been (implicitly at least, and often explicitly) structured to provide individualised health care and treatment to the better off, through the guidance of the physician.^24^ Although there was always a wider public health dimension (particularly in the management of epidemics), in Galenic medicine priority was given to diagnostic and prognostic skills rather than to the knowledge of (let alone production of) medicines. Often the cure was about diet, changes of air or life style, or recommendation of basic medical procedures such as bloodletting or purging, rather than ‘medicines’ as such – indeed there was no clear-cut category of a ‘medicine’ within such thinking (food and drink mattered as much as distinctively medicinal products). As we shall see, Maynwaring did not wish to relinquish this aspect of Galenic physic. Reinforcing this distinction was the social one which identified physicians as people who worked with their heads, based on superior education, to judge the course of action (and so were gentlemen), while other people did the manual work to manage the body or make the medicines. As Galenic physicians repeated, there was no such thing as a correct medicine for a disease as such, and medicines were at best dangerous tools, which could only be safely prescribed by an expert, whose chief skill lay not in prescribing the correct medicine but in knowing how and when to apply it (or not). Often they would prescribe combinations of medicines, to take account of specific circumstances, so that there could be no simple equation that medicine x had cured a case of y.

This model was challenged by the demand for ‘medicines’ to become precise and specific commodities associated with curing specific disorders. Physicians even promoted this tendency by using published pharmacopoeias to guarantee the standard composition of the medicines they prescribed – as patients associated physicians (despite their best efforts) with the efficacy of their remedies, they could not afford to let their quality slip. Moreover, humanist physicians prided themselves on using their learning to establish the ‘correct’ version of ancient medicines or to apply newly discovered drugs or plants. To offset the competition from chymical and empirical medicines, they sought to identify an authorised medical armoury, whose use could distinguish the orthodox from others. A growing minority of physicians wished to see effective chymical remedies included in that orthodox provision, notably the royal physician Theodore de Mayerne, who played the key role in establishing both the Society of Apothecaries in London under the supervision of the College of Physicians, and a royally authorised *Pharmacopoeia* of London. Published in Latin, and with its contents limited to the correct composition of each medicine (not stating what they should be used for) this was acceptable to orthodox physicians as a means to keep medicines, and hence apothecaries, under their control.^25^

Yet this tactic brought its own problems, as had become clear by the time Maynwaring commenced practice. As part of the radical attack on professional monopolies, Nicholas Culpeper translated the *Pharmacopoeia* into English and spelled out which medicines should be used with specific illnesses. If medicine largely comprised applying standard medicines, then surely that knowledge could be attained by an appropriate mixture of apprenticeship and book learning, as the ingredients were now public knowledge? Why did the public need physicians if they had standard medicines – surely patients, and certainly apothecaries who regularly dispensed them, could normally recognise the nature of the illness and prescribe the standard remedy? Indeed, if medicine consisted largely of medicines, then were not apothecaries, rather than physicians, the most obvious candidates to be general practitioners? This thought, originally posed by physicians as a ‘reductio ad absurdum’ to demonstrate that knowledge of medicines was the *least* important part of the physician’s art, came back to bite them when it began to be used by the champions of the apothecaries as a valid argument. And the more physicians tried to tighten their control over apothecaries to prevent them from ‘practising physic’ – that is diagnosing illness and prescribing medicines – the more they encouraged the apothecaries to adopt precisely this argument in garnering public support.^26^

Faced with this crisis, some physicians in Restoration London adopted a radical solution. The context was the heightening struggle between at least five groups: the London College of Physicians; a rival group of chymical physicians (including Maynwaring) who sought recognition for a Society of Chymical Physicians; other orthodox physicians excluded from the College; the Society of Apothecaries; and the burgeoning population of ‘empiricks’ offering their medicines and skills to the public.^27^ Amidst an increasingly acrimonious public debate, one line of argument that emerged envisaged a radical new model for physician leadership within a world of medicines. This was to propose that the proper and most important task of the physician was to experiment and perfect his own medicines and offer these directly to the patient, not send the patient with a prescription to the apothecary.^28^ The notion that each physician should experiment and produce his *own* medicines could certainly trace its roots back to ancient times (as its proponents stressed), especially if new knowledge of potential ingredients would justify experimenting with new combinations. But it was given great boost by the chymical physicians for whom it was a core principle from the start. Both Galenic and chymical physicians wished to bring this process of experimentation into a ‘professional’ setting, because both needed to distinguish themselves from mere ‘empiricks’ who (they asserted) relied either on trial and error or even simple fraud.

An extra dimension in England was provided by Baconianism, as championed by reformers during the mid-century crisis and then promoted by the Royal Society and other ‘virtuosi’ after 1660, with enthusiastic support from aristocrats who took up chymical and other experimentation.^29^ The ‘new science’ developed an ideology of experimentation and the pursuit of ‘useful knowledge’ over mere ‘words’, in part to overcome profound disagreements about theories and cosmologies, both in nature and in human society and government. In the 1650s more radical versions of this ideology had challenged the whole edifice of received learning and hierarchical knowledge (and the learned professions it supported), but the Restoration versions sought to tame and harness this critique by arguing that through ‘experiment’ a new synthesis could be developed that would allow useful knowledge to be generated without needing to undermine the social or political order. Within this model, the ‘rational physician’ like Maynwaring could re-establish his claim to authority by demonstrating that his education and mode of practice were not built simply on ‘words’ or received knowledge but on experiment, generating new and useful knowledge, not least in the production of new medicines. Hence he could distinguish his skills from those of the apothecary: in some versions the argument was combined with a withering critique both of the usefulness of the medicines within the standard pharmacopeia, and of the level of training required to be an apothecary. But how was such an experimental doctor, producing and selling his own medicines, to be differentiated from an ‘empirick’, ‘quacking’ his own medicines for sordid personal gain and without regulation? This was the dilemma which faced Maynwaring from the start of his London practice.

His DNB biographer (followed by the ODNB) noted that he was there by September 1663, when he published (in English) *Tutela Sanitatis* with an imprimatur of 25 September and a preface dated from his London study on 6 October, which informs readers that he will ‘provide advice and directions if applied to by letter or otherwise’ at his ‘dwelling next to the Blew-Bore on Ludgate Hill London’.^30^ However, they have missed two earlier publications which show him in London before May 1662. One is *Thesaurus Remediorum: a treasury of choice medicines internall and externall, exactly composed according to art, peculiarly and properly fitted and appointed against the infirmities of the principall parts of mans body …by Julius Degravere a learned physician …. The second impression revised, corrected and enlarged. The medicines diligently viewed, sealed up and duly ordered by the constant care and appointment of E.M. Doctor in Physick*. It has a Latin preface by ‘E.M. medicinae doctor’ dated ‘from his London study’ May 1662.^31^

I have now discovered an apparently unique copy of the first edition of this publication (unknown to the English Short Title Catalogue (ESTC) or Early English Books Online (EEBO)) in the library of the Royal Society of Medicine.^32^ The undated quarto has no title page but begins

Thesaurus Remediorum. Select and choice medicines (by an able physician, collected in his travels, and often experimented with great successe), being exactly composed according to art, carefully and properly fitted for the infirmities of the principal parts of the body. Reduced into small quantities, very neat and commodious for use and fitly proportioned and dosed for English bodies (by the assistance of an able English doctor), their virtues faithfully discovered and plain directions to use them, with set rates than none may exact; very cheap, that inferior people may purchase the benefit of them; and yet very efficacious for the diseases mentioned, whereby you may be your own Physician, and administer to yourself at a very little charge, as many have had the experience (very thankfully) in these infirmities.

It then has a three-column list of twenty-nine conditions (starting with convulsions and ending with plague) before continuing ‘by Julius Degravere …translated out of the Latine by an English Physician, assistant, and friend of the Authors for the publick good’. Although the second edition is considerably rewritten and enlarged (for example a substantial increase in the list of conditions treated, a new diatribe against ‘empiricks’ and considerably more detail on symptoms), it is clearly an expansion of the first and the ‘English physician’ of the first is surely E.M. in the second edition, who must be Maynwaring, since the sentiments expressed fit exactly with his later writings.

The remaining question is whether there was ever a Degravere (and, if so, how Maynwaring became his assistant, friend and translator), or whether Maynwaring chose to clothe his first appearance in print and as a purveyor of medicines under the cloak of editing and selling another man’s productions. There are no references in Everard’s later works to Degravere, or that he ever had a ‘mentor’, as many chymical physicians boasted of having. I cannot find any other references to the existence of a ‘Julius Degravere’.^33^ On the second page of the first edition there is a statement from ‘The Author to the Diseased’:

I have here comprised in a narrow compasse, what I purchased abroad by much study, travels, expence of time, and divers costly experiments; the cures of the principal diseases of the body according to the most approved artificial practice and certain experiments now used and famed in the most knowing parts of Europe: and have so contrived and reduced the medicines to such low rates that the poor may purchase health aswel as the rich; and for this I hope none will disesteem them, nor prize their worth by their price, but for the present will suspend their judgement and censure, until proof and trial, and then speak as you find.

Although phrased elegantly, such a statement contains all the elements typical of the advertisements of itinerant medics (often labelled quacks) who offered their services in London on the basis of extensive travel and experience around Europe but at competitive prices.^34^ Maynwaring later cloaked some of his publications purveying specific medicines in anonymity, so perhaps he felt the need to do so even more elaborately here. To maintain this anonymity, the medicines advertised (with prices, sealed with a coat of arms) could only be purchased via various London booksellers, not directly from the author. Although, as we shall see, some other graduate physicians, and even members of the College, advertised specific medicines they had developed, there is no surviving example of another graduate physician, even a chymical physician, advertising a complete set of medicines with prices. If this represented a bold entry into the London market (whose success we cannot gauge in the absence of any personal or business papers), the various measures to cloak (even falsify) his identity suggest that Maynwaring knew that this risked putting him beyond the pale from the very start of his London career.

However, in his *Tutela Sanitatis* (1663, reissued 1664) Maynwaring publicly committed himself, both to the same armoury of medicines (listed with prices and directions, but not ingredients), and also to medicines as the distinguishing mark of the excellent physician. Explaining why he did not publish the ingredients, he argued that the making of:

medicines does not belong to you, of other employments, professions, trades, or who ever not authorized in the faculty; this belongs to the Physitian to know and appoint, as his propriety, and you to have the benefit and use of them, nor ought any to challenge the making of medicines as his right, but as subordinate, a servant of Physitians, to do the toyling part and servile work that belongs to it. …And since medicines is become a trade, it is a trade of the greatest and most general concernment I know. And I must say, an error, mistake or abuse in the medicine is far greater and more dangerous, then a deficiency or error of the Physitian in his judgement of the Patient. For a good medicine is not so tyed up and restrained to one disease, But it shall operate for good in many others, (seasonably given in due quantity) so that if a Physitian do not so exactly determine aright concerning the Patient; yet if the distemper he imagines, have but an affinity and proportion with that which really afflicts the Patient, and he gives a proper medicine according to his own determination, his medicine shall prevaile and succeed well. But an adulterate bad medicine, though given by the most skilful hand and deliberate consultation, shall have bad effects: and therefore I may affirme, that a Physitian of ordinary parts, with extraordinary curious medicines, shall performe more and greater cures, and have less miscarriages, then the most knowing and learned, with ordinary, sophisticate medicines. And I think it much more necessary, that the Physitian should look into the medicine then the chamber-pot, as a thing of greater concernment, and he shall practise with more security to his own reputation, and less hazard to his patients life. And that Phisitian who spends some time in Pharmacy shall finde more satisfaction in seeing a medicine duely prepared and compounded once, then in reading it a twelvemonth.^35^

He proceeded to critique the quality of the ingredients and composition of most standard medicines, before concluding ‘from hence may be collected the reasons and motives which first put me upon this work, and made me a Pharmacopoeian to my own practise’:

I was moved to communicate, and convert my private stock to a publick store, for the benefit of those who have not a fee ready, suitable for a Doctor but must apply themselves to bold professing Empiricks, and other pragmatical fellows that deserve to have their ears cut for their impudent ignorance in the practice of Physick, and saucie usurpation of so high and mysterious employment that the most learned men of the faculty in all ages have, and are still in the disquisition; the many abuses whereof in Medicines, and their improper use, is now the greatest and most dangerous Cheat in this age:^36^

He developed the same theme in his *Morbus Polyrhizos et Polymorphaus*, published in 1665 but licensed 9 September 1664:

The greatest deficiency I have observed, in some, though otherwise sufficiently stockt with Learning and accomplished, is in Medicines…. I must affirm that an expert knowledge in the Pharmacopoietical part of Physick, do as much belong to a Physitian and is so necessary, that without it he cannot be said to be compleat; for, he that is not an Artist herein cannot direct and correct as he ought, by the promptings of a bare contemplative knowledge: and although he excludes himself from inspection into the practick part, as an unnecessary trouble and below the dignity of his title, yet he is not excused thereby, but his reputation payes for the miscarriages and abuses therein.^37^

I have quoted these passages at length because they predate the publication usually cited as beginning the argument for the experimental physician, namely *A Letter concerning the Present State of Physick* (1665) by ‘T.M.’, which only received its imprimatur on 30 March 1665, although that work introduced a much broader range of issues with its vision of how the College of Physicians might promote and institutionalise experimental medicine within the education and careers of the physician.^38^ Cook’s description of Maynwaring’s 1668 work, *Medicus Absolutus*, as ‘echoing’ T.M. by proposing ‘physicians all make their own drugs with their own hands …a solution first proposed by T.M.’ overlooks Maynwaring’s earlier writings.^39^ However, in Maynwaring’s case it seems that this argument for experimental medicine, while no doubt sincerely maintained (and possibly even instrumental in attracting him to chymical medicine in the first place), also formed a crucial means to justify the sale of his own proprietary medicines with set prices, in a fashion more akin to an ‘empirick’ than a regular physician. It was perhaps for this very reason that in these and his later writings he felt the need to distinguish the grounds of his practice so strongly from such ‘empiricks’ (or indeed from practising apothecaries). Maynwaring’s ‘compleat physician’ required academic learning and rational argumentation and was always a gentleman, only reluctantly engaged in the ‘trade’ of medicines to serve the public, who should not ‘toil in the drudgery’ of chymical production with his own hands, but rather supervise the work of his servants.^40^

Over the next few years, Maynwaring established himself in the circle of London’s chymical physicians, earning respectful references from Edward Bolnest and Thomas Sherley.^41^ He himself praises Bolnest and his ‘friends’ George Starkey and George Thomson, while Thomson praises Maynwaring, criticising those who could portray such learned men as Maynwaring (and himself) as ignorant ‘empiricks’.^42^ Unlike Thomson, Maynwaring did not get drawn into any direct polemical exchanges,^43^ although one critic (probably Henry Stubbe or a physician collecting information for him) made notes on Maynwaring’s work for a defence of Galenic physicians, observing ‘I suppose Mr Boyle, Maynwaring and Merrett set out books like bills on posts to tell where you may have arcanas’.^44^ Like other chymical physicians, Maynwaring sought court patronage, exploiting the popularity of chymical experimentation with the King and many aristocrats. The second edition of his *Tutela Sanitatis* in 1664 was dedicated to Prince Rupert, his *Morbus Polyrhizos et Polymorphaus* to the Earl of Lindsey and then *Solamen Aegrorum* in Latin to the Duke of Buckingham ‘the great Maecenas of chemists’ in May 1665.^45^ Lindsey and Buckingham (and Kenelm Digby) supported the proposal for a Society of Chymical Physicians published in March 1665, to which Maynwaring was a signatory as one of 34 chymical practitioners.^46^

London was then attacked by plague, which the chymical physicians hoped would demonstrate their superiority over Galenists both in their medicines and in their devotion to duty, as they remained in London while most College physicians left town with their elite patients. Maynwaring’s house on Ludgate Hill was given as one of those where the public could obtain chymical medicines against plague in a leaflet dated 28 June 1665.^47^ He was given control of the pesthouse run by the Governors of the Incorporation of the Poor of Middlesex, to whom (on 14 December 1665) he dedicated his next work, *Nova Medendi Ratio* (1666, but licensed 12 December 1665).^48^ He claimed in this dedication that 56 of the 80 patients he had cared for in the pesthouse had recovered, and a report of his on the plague survives.^49^ Giving evidence on the workings of the Incorporation before the Commons in 1671, its Treasurer reported:

In 1665 the Plague broke out, and a covenant was, that the Corporation should maintain all that have any pestilential disease, or be disabled …they bought a house for the sick, and they agreed with a Doctor for every person recovered of the Plague, 20s for physic, &c, and nothing if he died; one Doctor went away, but another staid and did his duty. The Plague was so fatal, that he called for money, or he must let all out to be destroyed. Several sums of money were furnished him, but none from the public. Fifty-six persons were actually visited, and actually recovered, and the Doctor had his money, but some time after.^50^

This pesthouse (and Maynwaring’s role) has gone unnoticed by London’s plague historians.^51^ He probably led the procession on 19 September 1665 described by Dr Simon Patrick:

I saw last Tuesday about 30 people in the Strand, with white sticks in their hands, and the dr. of the pest house walking in his gowne before them; the first woman rid on an horse, and had a paper flag on the top of her stick with Laus Deo written in it. They were going to the justices, being poor people sent thither and recovered by him of the plague. He seemed to take no small content in his stately march before them.^52^

If this was him, Maynwaring was clearly keen to advertise his curative abilities very publicly indeed, while his agreement to a ‘no cure, no fee’ contract would also have been objectionable to the College.^53^

In 1666 he published a second edition of *Morbus Polyrhizos* (also licensed 12 December 1665) and moved house, following the Fire’s destruction of Ludgate Hill. His next work (on consumption) *Tabidorum Narratio*, (1667, but with an imprimatur of 13 October 1666), was prefaced from ‘my house in Clerkenwell Close’.^54^ He was also called ‘gent of St James Clarkenwell’ when, as a bachelor ‘aged 35’, he was licensed to marry Anne Ayloffe (spinster aged 26) at St Bartholomew’s the Less or Gray’s Inn chapel on 31 December 1666.^55^ His marriage suggests he felt confident that he had established himself as a leading chymical physician. From 1667 to 1671 his publications are dated from Clerkenwell Close, before he moved back to the Fleet Street and Strand area (Fetter Lane, then Wine-Office Court in Fleet Street in 1675, then various houses close to the Inns of Court, then Denmark Court) for the rest of his career.^56^

In 1668, along with further editions of his works on scurvy and consumption, Maynwaring published perhaps his most ambitious book, *Medicus Absolutus Adespotos, or the Compleat Physician* (imprimatur 27 February; dedication dated 8 March), which justified physicians composing and prescribing their own medicines as a necessary return to the ancient method of physic from the corruption of modern practices.^57^ This argument was developed in ‘The Ancient Practice of Physick Revived and Confirmed’, forming the second half of *The Pharmacopeian Physician’s Repository* (1669) and then in *Praxis Medicorum Antiqua et Nova* (1671, licensed 17 March, with preface from his house in Fetter Lane).^58^ He distinguishes various types of practitioners. His severest criticisms are of ‘practising apothecaries’ (apothecaries who offered medical advice) and ‘chymical empiricks’ whose purely empirical methods and fraudulent claims he denounced as strenuously as any College physician might.^59^ Unlike some of his fellow chymical physicians he never praised empirical medicines in comparison with Galenic ones, and explicitly rejected Marchamont Nedham’s argument ‘that there should be a liberty allowed in the profession of physick’, which he predicted would see ‘a monstrous brood of illiterate practisers’ as ‘the whole profession would fall into the captivity of rude, mechanic invaders’.^60^

His second category of physicians comprised ‘rigid Galenists’, Galenic physicians who rejected chymical medicines and justified their practice by classical knowledge. Despite his insistence on academic learning, Maynwaring is dismissive of this slavish adherence to the ancients, arguing that the true ancient physic was experimental and progressive: proper adherence to the ancients involved following their experimental method to discover new truths, not sticking to their theories or specific methods of treatment.^61^ As a chymical physician, he rejected the humoral model of the body, as it related to cure, although his account of the non-naturals and preventative medicine was compatible with the model of the four constitutions,^62^ and he (unlike Thomson) never rejected outright all Galenic remedies, recognising circumstances in which bloodletting, for example, might be appropriate.^63^ His main objection to contemporary Galenism was its ‘pen-prescribing’, that is, observing the patient and writing a specific prescription for a particular case, based on a combination of medicines, which was then delivered to the apothecary to prepare and administer.^64^ Not only did Maynwaring not trust the apothecary to prepare the medicine properly (sharing the critique of apothecaries’ standards of practice common to other physicians in this debate),^65^ but (distinctively) he argued that it was theoretically and practically unsound to create such compound prescriptions. To do so, he claimed (in a dramatic reversal of standard Galenic arguments), was to gamble with the life of each patient by creating an untested medicine, since there was no experimental proof that each specific combination was going to be effective. Even if the individual ingredients were efficacious (and Maynwaring did not think most Galenic ingredients were as effective as chymical ones, objecting to their rough and unpleasant characteristics), combining them would produce a substance with unknown properties. Furthermore, by mixing ingredients and leaving their administration to the apothecary, the physician could never observe rigorously the efficacy of his remedies.^66^

The third category of physicians, the ‘Galeno-chymists’, were those who accepted the efficacy of chymical remedies and were prepared to include them, even rely largely on them, in their practice, but did not make their own medicines and prescribed like the Galenists.^67^ This was the approach taken by those in the College of Physicians who, in fighting off the challenge of the Society of Chymical Physicians, argued that they had integrated into their practice the best of chymical medicine and were themselves well informed about chymistry (and even more about anatomy), while rejecting Paracelsian or Helmontian theories and their critique of traditional practice.^68^ Again, Maynwaring was less polemical than other chymists in questioning this approach, but he was clear that, by failing to put experimental medicines at the centre of their practice, such physicians would never be at the vanguard of medicine. Anatomy, while useful, did not address the key workings of the body which determined the cause and course of disease.^69^ Only the physician who experimented, both to create and test medicines, could truly claim to know what he was prescribing and to advance medicine by developing new and more effective remedies. To do this, the physician would need to break with traditional modes of practice, limiting his clientele to a manageable number. Having met each patient, he then needed to return to his laboratory and ponder each case, considering which of his armament of medicines would best treat it. He should then observe the outcomes and modify and develop his medicines for future cases.^70^

The practitioner who followed this practice was Maynwaring’s fourth type – the ‘compleat physician’.^71^ In describing this figure, Maynwaring was in close alignment with other chymical physicians, and George Starkey was probably his role model.^72^ But, unlike Starkey, Maynwaring never got drawn into the specifics of chymistry, showing no interest in alchemical traditions or secrets, at least in his publications. Admittedly, this may be misleading, because Maynwaring was very clear that it would be inappropriate to publish the detailed composition of his medicines: only trained chymists who knew how to make medicines properly should know their contents, and they should apply to him privately for information. The experimental physician was entitled to the profit of his own inventions, and should keep details of their composition away from ‘empiricks’ who would pervert and exploit them (while not conducting the ongoing trials and improvements of medicine that were required). All his books are aimed at informing and persuading the public or other types of practitioner, not his fellow chymical physicians, so they were not contributing to an experimental debate *within* chymistry.^73^ Yet this indicates that Maynwaring’s own priorities were always medical rather than chymical – he saw himself primarily as a ‘compleat physician’ not a chymist.

This conception is reflected in his two new publications in 1669, *Vita Sana et Longa* (licensed 4 August)^74^ and his *Pharmacopeian Physician’s Repository*.^75^ The former was a much expanded version of *Tutela Sanitatis*, offering a guide to preventative medicine organised around the six non-naturals, with particular emphasis on regulation of the passions; a later version was published in 1683 (preface, 1 November 1682) as *The Method and Means of Enjoying Health, Vigour and Long Life adapting Peculiar Courses for Different Constitutions, Ages, Abilities, Valetudinary States, Individual Proprieties, Habituated Customs and Passions of Mind.* ^76^ However, while *Tutela* had included a therapeutical section listing Maynwaring’s ‘magazine of medicines’ (with prices), these were now separated out into the separate *Repository* (although the copy digitised in EEBO has both texts bound together, so they were probably sold as a pair).

However radical his therapeutic practice, Maynwaring maintained a commitment to the traditional role of the physician as an advisor to individuals on preventative medicine.^77^ Nor was this at odds with his chymical philosophy, for Maynwaring considered that the most important determinant of health was ‘the soul’ or vital spirit (he only occasionally uses the Helmontian term ‘archeus’).^78^ Throughout his writings, but expressed most directly in his 1692 *Monarchia Microcosmi: the origin, vicissitudes and period of the vital government in man*,^79^ Maynwaring adopts a very strong distinction between an active vital principle, spirit, soul or governor of the body, and the passive machine of the body itself: rejecting the triple model of vegetable, sensitive and rational souls, or the language of animal spirits, in favour of the sole sovereignty of a vital governing agent.^80^ In politically charged language, Maynwaring stresses the monarchy of this vital agent, portraying ill-health as the result of either invasion from outside or rebellion from within, and the need to re-establish the proper monarchical government, whether through correct regimen or the effective use of his armoury of medicines. He underlines this military metaphor in the subtitle of *Tutela Sanitatis*: ‘Bellum necessarium sive medicus belligerans the military or practical physician reviewing his armory: furnished with medicinal weapons and munititions against the secret invaders of life; fitted for all persons and assaults’. These medicines should assist the proper monarch to reduce the body back to its proper passivity, subject to the governing agent.^81^

Maynwaring’s approach to medicines follows from this Helmontian model, seeking to strengthen and support the vital governing agent in managing all parts of the body, and expelling ‘peccant or morbific humours’ whose resistance to correct government underpinned all disease. Throughout his career, Maynwaring expressed a strong preference for using a few ‘catholic’ or ‘universal’ medicines which were capable of restoring the whole body, by working through the agency of the vital spirit. Facing the inevitable charge that he was seeking a ‘panacea’ (like some ‘empirick’), Maynwaring responded that, just as the food processed by the stomach operated throughout the whole body, so a well-chosen medicine could be applied by the vital spirit to any malfunctioning parts of the system.^82^ Rejecting the standard humoral system, he recognised the multiplicity of chymical agents creating potentially morbific humours, yet regarded them as all susceptible to a few medicines designed to break down obstructions and promote evacuation, which he believed his chymical medicines could achieve in a gentle and selective manner, so preventing the many dangerous forms of illness caused by standard purgatives and other Galenic treatments.^83^ Although his earliest works list 40 or 50 medicines, directed for use at diseases of different parts of the body,^84^ by his scurvy writings in the mid-1660s he had reduced his anti-scorbutic medicines down to two: ‘scorbute pills’ and a ‘Catholic elixir’, stating that ‘an able practised physitian, rightly principled in the nature of diseases, and expert in pharmacy, may well contract his practice within the compass of a few medicines …endowed with a large portion of universality, bringing within the latitude and circle of their energy very many diseases, restoring the faculties decayed, the great engins of our bodies; roborating [strengthening] the *primum mobile instrumentaliter*’.^85^ In *Nova Medendi Ratio* he further explains that ‘the three grand intentions of cure are purgation, transpiration and roboration’, and medicines that operate ‘radically’ under one of these headings are better than specifics. He has improved his ‘scorbute pills’ so they can now operate for purgation in all persons and cases, developed a ‘sudorifick extract’ for transpiration (his main medicine against the plague), and his ‘Catholic elixir’ for roboration, though he does not rule out using other medicines in special cases.^86^

In his *Medicus Absolutus* he expands on his discussion of types of medicine, distinguishing ‘Catholic, Specifick and Appropriate’.^87^ A catholic medicine is a medicine ‘curing all diseases in all persons’ or at least, as he later qualifies, ‘of necessary use in all diseases’, and ‘a plurality of them is allowed’. A specific medicine is ‘proper and peculiar to the cure of one disease only, and in all persons’. An appropriate medicine is ‘adapted to one individual person, for this or that disease or complicated diseases, designed for his case alone’^88^ which, as discussed above, he claims is untested and much inferior: ‘Take a true catholick known medicine, and ten peculiar appropriate medicines for the same operation, never tryed in the persons for whom they are appointed; and you will finde that the catholick medicine (if true and not counterfeit) shall better agree, have less miscarriages, and take better effect in the ten several persons, then the peculiarly *appropriate* medicines for each.’ He offers to undertake a trial between his catholic medicine and ten such ‘appropriate’ medicines for any single operation ‘whether it be purging, sweating, strengthening, &c’ to see which performs best.^89^ Although, as this quotation suggests, he still seems to have three main operations of medicine in mind, he then suggests six main types: ‘*Cathartick, Diaphoretick, Diuretick, Anodyne, Bezoardick,* and *Restaurative’.*

The first cleanseth and evacuates by stool; the second by or through all the pores of the body; the third by Urine; the fourth mitigates pains, allures the *Archeus* to rest, and bridles his *exorbitant* motions, which are many and frequent; the fifth resists malignity and venenous assaults; the sixth *roborates* and restores the *vital principles.* And these are the *grand* and chief *classes* of *medicinal* operations, wherein a *compleat pharmacopoeian* physitian forms his medicines and labours by various experiments and gradual Improvements, that they may be *adequate* and answer the full intent of each c*lassical* distinct operation in all bodies: and such being made *catholick* and *radical,* are standing ... And the reason why his *indications* for cure are fewer is, because his medicines are radical, and respect the vital and fundamental principles *primò intentionalitèr;* not humors, temperaments, qualities, and the various *phaenomena’s* or *symptomes* of diseases from thence; save only *consequentèr*.^90^

The implications of this final point are followed though in his later writings, which, with increasing emphasis, distinguish between the underlying causes of diseases and their symptoms, accusing Galenic medicine (and other experimental physicians) of treating symptoms rather than causes, often with medicines which then weakened and even killed the patient. This approach underlies his *History and Mystery of the Venereal Lues* (1673, licensed 18 November 1672),^91^ and more explicitly *The Frequent, but Unsuspected Progress of Pains* (1679, licensed 5 September 1678), as well as his *Ignota Febris* (initially 1691, much expanded in 1698), a natural culmination since it was the treatment of ‘fevers’ above all that represented to Maynwaring the folly of regarding what was merely a symptom of disease as a disease to be treated in its own right.^92^ The matter is discussed most systematically in his *Inquiries into the General Catalogue of Diseases* (1691).^93^ Here Maynwaring rejects the division of diseases into ‘similar, organical and common’^94^ proposing instead a distinction between ‘spirital’ and ‘corporal’ diseases, the former involving a cause that predominantly affects the ability of the vital spirit or principle to govern the body properly, while the latter predominantly involve ‘undue confirmation and constitution of the visible corporal parts’. But in both cases

the vital *Spirit* being thus variously provoked and afflicted; begets or forms various diseases, from the diversity of *organs* used, and *functions* to be performed thereby. We may hence learn, that diseases in their multiplicity, and variation by denomination, from *parts* affected, and *functions* impedited; do not lye so wide asunder, and differing, as the world does imagine; since the chief moving principle, or obstructed in motion; is one and the same in all the faculties, but irritated to disorder, or impedited in vital government.^95^

Hence Maynwaring was constantly attracted to the notion of a single universal medicine. In 1675 ‘E.M.’ published *The Universal Scorbutick Pills, and Radical Purifier of Nature. Operating by purgation and urine, with the greatest ease and success in various diseases and infirmities*. ^96^ This had demonstrated that ‘*physicians* of the greatest *fame,* as they were diligent in the *preparation* and *tryals* of medicines; so were they ambitious their *names* should give *title* to medicines of their own invention and industry’. Although

*Illiterate* men of *mean* and *broken* fortunes, (taking advantage upon this *absurd* neglect of the *professors*) have *craftily* and *gainfully* obtained the custom of setting forth their trivial slight medicines, so that some *worthy* physicians now *reviving* the *primitive* practice, are at present discouraged from offering the *products* of their a*rt* to the world, for fear of *scandal,* and being accounted in the number of those quacks. But the *publishing* of medicines thus shamefully *defamed* by *ignorant* pretenders, must be restored into esteem by the *learned,* which will prove most *advantagious* to the people: and it is their interest to *incourage* skilful experienced a*rtisti,* that they may not *lock* up their *rare* inventions, and *fortunate* experiments, confining them within the narrow compass of a *private* practice; but rather that they expose their *arcanum* to publick use, that all who stand in need may partake thereof…Physicians then will be stimulated to *outvye* and *excell* each other in the *rarity* and *excellency* of their remedies, being to undergo a publick tryal. And when men of the greatest *knowledge* and *experience* shall thus produce their comprehensive *catholicks* and appropriate *specifick* medicines; a *treasury* will be laid open, and what is most *excellent* and *rare,* will be known and conveyed through the *nation,* for the *ease* and *relief* of the Infirm and languishing.^97^

Until this point in the mid-1670s, Maynwaring may well have hoped that the vision he portrays above might still happen, allowing a ‘compleat physician’ to produce and sell proprietary medicines, while still practising and being recognised as a learned physician among gentlemen. But over the next fifteen years the prospects for maintaining this balance deteriorated. The College resumed prosecution of a range of practitioners for illegal practice, including advertising proprietary medicines, and disciplining their own members for similar offences. Particularly affected were five College physicians who, in 1687, responded by creating their own joint practice, making and dispensing their own medicines, and justifying this in a publication, *The Oracle for the Sick*, which also offered patients guidance in diagnosing symptoms in their bodies. The two Englishmen among the five, Richard Browne and John Pechey, were both in trouble with the College for advertising their own proprietary products – Browne’s ‘London pills’ and Pechey’s ‘purging pills’ – as well as translating and abridging medical works for a broad audience, something which Pechey continued to do, notably with the works of his friend Thomas Sydenham and his 1694 *London dispensatory, reduced to the practice of the London physicians wherein are contain’d the medicines, both Galenical and chymical, that are now in use. Those that are out of Use are Omitted: And such as are in Use, and not in the Latin Copy, are Added; with Vertues and Doses.* ^98^ Further complicating matters, the College itself began (from 1687, though it took a decade to implement) to consider setting up its own dispensaries to undercut the business of apothecaries who were acting as physicians, yet in doing so they eschewed the argument that each physician should produce his own medicines, preferring instead to require physicians to dispense according to the official pharmacopoeia. There is no space here to explore all the complexities of this process, but it must have become increasingly clear to Maynwaring that his ‘compleat physician’ would never be accepted. Perhaps for this reason, his later publications (many of which are short pamphlets clearly aimed at a non-medical readership) begin to appeal more directly either to the ordinary consumer, or to the state as a potential patron, using utilitarian arguments rather than appeals to medical history or theory.

In 1684 ‘a strict examiner of medical art’ published *The Catholic Medicine, and Soverain Healer rectifying and assisting the Depraved Functions, of Infirm and Diseased Bodies.* ^99^ Though anonymous, this is clearly the medicine identified as his own in his later writings.^100^ Unusually for him, Maynwaring refers directly to his own health in justifying this medicine, stating ‘The *author* or *inventer* of this medicine, was the *maker* of it; and also the first *patient* that try’d it upon his own body.’^101^ Struck ill, ‘I bethought myself of a medicine, which long before I had begun to form the designment; and had noted it in my papers, intending at some leasure times to review and perfect the designe thereof’. This ‘*extract* performed all the requisite offices belonging to this great *cure:* It acted alone the various *operations* of divers medicines, which successively and methodically are used in such cases. This single medicine, but once in twenty four hours taken, and sometimes but once in forty eight hours, performed the whole work, and suppl’yd the place of a numerous train of medicines.’^102^ Then, ‘I was desirous of seeing tryals upon *others* in different cases, to confirm the opinion I had of this *medicine;* and therefore I waved the use of other proper and good medicines, for peculiar *operations* and purposes, in the cure of several infirmities and diseases, in divers persons, and prosecuted only with this *Catholick Extract;* from which successful experiments and proceedings therein, I am further informed, and fully satisfyed of the *worth* and *usefulness* of this *medicine,* both in *young* and *old,* and of both *sexes,* variously diseased’.^103^ He claims it ‘is a strange compound indeed, as ever was; but it is a true one; and the best (if I may be Judge) that ever was yet; at least, the best that I ever saw, or can hear of; and I have had the view of thousands, upon inquiry and search for many years, in print and manuscripts: besides my own *experiments* and *tryals,* not a few, for satisfaction’.^104^ In particular ‘this *medicine* doth appear the most commodious medicinal provision (as *ready, durable,* and *portable*) for *armies, navies,* and *hospitals:* compleated for all emergent occasions, and the most efficacious means that can be used; for *wounded, contused, apostemated, ulcerated, maimed* persons, and sick people; nothing superior, nor equal to it now in practice’.^105^ Hence ‘to conceal it, were a *crime;* and to confine this most useful medicine to a private practice only; were against the rule of charity’.^106^

From 1689, Maynwaring became ever more focused on this ‘catholic medicine’ and its potential value in the war against France. Although the main text of his *A Serious Debate, and General Concern, relating to Health and Sickness* still promotes his antiscorbutic pills and restorative elixir, a postscript focuses on his ‘single medicine .. [which] will exceed all medicines, as yet found out …the most commodious, expedite and powerful means, preventive and curative, for army and navy’.^107^ He expands on this in 1690 in his *The Test and Tryal of Medicines and the Different Modes of Medical Practice*.^108^ He breaks off his discussion of disease types in his *Inquiries into the General Catalogue of Diseases* to urge the authorities to adopt his medicine as the basis of provision for ‘fleet, army and hospitals’, criticising with increasing bitterness the failure of those commissioned to provide medical supplies, not only for wasting £10 000 on useless medicines but also thereby wasting the lives of 10 000 soldiers and sailors.^109^ As Cook has highlighted, the growing demand of the armed forces for empirically proven medicines addressing specific complaints was challenging Galenic orthodoxies and making the state less and less sympathetic to the monopoly claims of the College, whether it was in relation to physicians like John Colbatch and William Cockburn, or to the medical provision offered by surgeons and apothecaries.^110^ However, there is no evidence that Maynwaring succeeded in bringing his medicine to the attention of patrons in the army or navy.

Finally, he brought together his theoretical writings and his advocacy for this particular medicine in his tract *The Mystery of Curing Comprehensively Explained and Confirm’d, by Exemplar of the Catholic Medicine* (1693, licensed 11 January).^111^ Maynwaring concludes thus, ‘I have viewed thousands of medicines in *pharmacopoeia’s,* practical *authors,* and *manuscripts chymical and Galenic;* and took observations upon their failings…And until *Nature* produceth a new *materia medica;* I believe we shall not be *blest* with a *better* medicine…’.^112^ By 1698 Maynwaring was calling this medicine ‘a Catholic purifying extract’ which ‘operates by *Stool,* and *Urine;* sending forth by these *Canals* of Emission; all morbific vitious Matter, that must pass those ways, opens *Obstructions* of the *Spleen, Liver, Pancreas, Mesentery, Kidneys,* &c’. It was one of two comprehensive medicines, along with a ‘*Sudorific* Medicine’ which ‘opens all the Pores, to breathe out Impurities that infest the *Habit* of Body, and external Parts: Clears and takes away all Cutany Defoedations, *Spots, Scurf, Scabs, Pustules, Tettars, Itch,* &c’. He promised ‘I will ingage to Cure more *Fevers* with these two Medicines only; than any of you shall with the two *hundred* Medicines appointed by *Riverius,* in his Practice upon *Fevers*’.^113^ Sadly for Maynwaring, neither the state nor the public appear to have accepted his claims. His ‘Catholic extract’ was presumably the medicine he vainly requested the College to approve in 1701, as he advertised it in the *Flying Post* that year as ‘The Purifying Extract’, available from ‘the author’s house in Denmark Court in the Strand’ and ‘confirmed by divers successful proofs in long practice’, summarising it as ‘a catholic expedient, the most hopeful radical means against contumacious chronic diseases and various acute sicknesses’.^114^

As we have seen, the years to his death in 1713 seem to have been marked by growing obscurity, reduced to advertising cures ‘by means extraordinary’. Eventually, Maynwaring had been defeated by the difficulty of presenting the development of experimental medicines (and especially comprehensive ones) as the mark of the ‘compleat physician’, rather than of the ‘empiricks’ whom he had always regarded as his enemies. For a period in the 1660s and 1670s the struggle between physicians and apothecaries, together with the vogue for experimentalism in elite culture, fostered both at court and in the Royal Society, had given the notion of the experimental physician, making his own medicines, a substantial attraction to a range of physicians (not just chymists) who wished to sustain a model of learned physic on a new foundation of scientific progressivism. Maynwaring’s publications show him as one of the earliest, most eloquent and persistent of those advocating this approach, with a learned grasp of medical history to support his case, and without the personal polemic that vitiated many other contributions. But, unlike those on whom Cook and Wear have focused, Maynwaring clearly had a further purpose, namely to use the idea of the ‘compleat physician’ to justify his promotion of his proprietary medicines while avoiding the label of ‘empirick’ and maintaining a genteel practice as a learned physician. Yet, even during this period, it is questionable whether Maynwaring’s willingness to publicise for sale his magazine of medicines was acceptable professionally: hence his various strategies of anonymity or editorship. Equally his urge to identify a few ‘Catholic’ or universal medicines, however persuasively he argued for their rational foundation, must have reinforced suspicions of his trustworthiness. Like other chymical physicians, he was not acceptable to the College (though there is no evidence that they attempted to hinder his practice), and while many chymical remedies were absorbed into the orthodox pharmacopoeia, his remedies were not. For the time being a rational method of diagnosis had proved a more acceptable foundation for the learned physician than experimental medicine.

## Footnotes

1.
See Harold J. Cook, *The Decline of the Old Medical Regime in Stuart London* (Ithaca, NY, and London: Cornell University Press, 1986), 141–82; Harold J. Cook, ‘Physicians and the New Philosophy’, in Roger French and Andrew Wear (eds), *The Medical Revolution of the Seventeenth Century* (Cambridge: Cambridge University Press, 1989), 246–71; Andrew Wear, *Knowledge and Practice in English Medicine, 1550–1680* (Cambridge: Cambridge University Press, 2000), chs 9–10; Dmitri Levitin, *Ancient Wisdom in the Age of the New Science* (Cambridge: Cambridge University Press, 2015), 274–86. The same debate has recently been explored from the perspective of another participant by A. Mauck, ‘ “By merit raised to that bad eminence”: Christopher Merrett, Artisanal Knowledge and Professional Reform in Restoration London’, *Medical History*, 56 (2012), 26–47. Merrett also failed to maintain his status as a learned College physician.10.1017/S0025727300000260PMC331489623752982

2.
Antonio Clericuzio, ‘Maynwaring, Everard (b.1627/8)’, *Oxford Dictionary of National Biography*, Oxford University Press, 2004 [http://www.oxforddnb.com/view/10.1093/ref:odnb/9780198614128.001.0001/odnb-9780198614128-e-18449?rskey=xG7cxj&result=1]. Although my article supplies more accurate details of his career (including his marriage and death), Clericuzio’s biography accurately summarises his key intellectual positions. I have followed his standardisation of the name.

3.
Levitin, *op. cit.* (note. 1), 284–6, 328; S. Pender, ‘Seeing, feeling, judging’, in Jan Frans van Dijkhuisen and Karl Erenkel (eds), *The Sense of Suffering* (Leiden: Brill, 2009), 477–513; S. Pender, ‘Subventing disease’, in Jennifer Vaught (ed.), *Rhetorics of Bodily Disease and Health in Medieval and Early Modern England* (Farnham: Ashgate, 2010), 193–218: 212–14; Katherine Walker, ‘Pain and Surgery in England *c*1620–1740’, *Medical History*, 59 (2015), 255–74: 260–1; Margaret Delacy, *The Germ of an Idea* (Basingstoke: Palgrave, 2016), 44–6. Peter H. Niebyl, ‘Science and Metaphor in the Medicine of Restoration England’, *Bulletin of the History of Medicine*, 47 (1973), 356–74: 358–9, 364–6 and 373, treats Maynwaring as a Helmontian in his response to Galenic views of digestion and other aspects of the body.

4.
Everard Maynwaring, *Ignota Febris: Fevers Mistaken in Notion & Practice*, 2nd edn (London: 1698) Wing M1495, 153. The original edition of this title in 1691 (Wing M1494) was only 8 pages long. Hereafter publications by Maynwaring are given without his name, with Wing details when publication is discussed: they are published in London unless indicated.

5.

George Clark , A History of the Royal College of Physicians, Vol. 2 (London: Clarendon Press, 1966), 456.

6.

*Post Man*, 31 January 1702 and 14 March 1702 (Daniel Brown) and 8 October 1702 (William Lucas, with ‘event’ not ‘events’ in title). This edition is not recorded in the English Short Title Catalogue (ESTC).

7.
On 19 August 1694, one Ann Halford from ‘Dr Manwarings of Grays Inn Lane’ was buried in St Andrews Holborn, so he had presumably lived here both before and after his period at Denmark Court.

8.

C.H. Josten (ed.), Elias Ashmole, Vol. 2 (Oxford: Oxford University Press, 1967) 320–1, 324, 338, 454, 479, 688.

9.

Arnold G. Matthews , Walker Revised (Oxford: Clarendon Press, 1948), 221.

10.

John Venn and J.A. Venn , Alumni Cantabrigienses, Vol. 3 (Cambridge: Cambridge University Press, 1924), 127 It is hard to reconcile this age, making him born in 1627–8, with the statement on his marriage licence in December 1666 that he was aged 35 (see note 55), or on his engraved portrait in 1668 when he is described as ‘aetatis suae 38’ (*Vita Sana et Longa* (1669), frontispiece). An earlier account of his matriculation (*Notes and Queries* 3rd ser. 2 (1862), 506) states it was on 21 January 1645 and he was 16, while another article in *ibid*., 3 (1863), 198 quotes a family manuscript stating he was born in 1629. There are no Gravesend registers for this period.

11.

*The Frequent, but Unsuspected Progress of Pains, Inflammations, Tumors, Apostems, Ulcers, Cancers, Gangrenes, and Mortifications Internal therein* (1679) Wing M1492, 142. Revised edns were published as *Pains afflicting Humane Bodies their Various Differences, Causes, Parts Affected, Signals of Danger and Safety* (1682) Wing M1509 and *Latent Dangerous Progress and Fatal Events of Internal Pains* (1702: see n. 6).

12.
Matthews, *op. cit.* (note 9), 221.

13.

*Frequent, but Unsuspected Progress*, *op. cit.* (note 11), 105, 111.

14.

*Morbus Polyrhizos et Polymorphaeus. A Treatise of the Scurvy* (1665) Wing M1500 with various 2nd edns (1666) Wing M1500A, M1501 and M1502, 3rd edns (1669) Wing M1503. M1503A and M1503B and fourth edns (1672) Wing M1504 and (1679) Wing M1505.

15.
BL Harleian MS 2094.

16.

*Ignota Febris*, *op. cit.* (note 4), (1698), 155; *A Serious Debate, and General Concern, Relating to Health and Sickness*, second impression (1689) Wing M1512A, 8.

17.
In the expanded 1669 edn of his *Morbus Polyrhizos*, *op. cit.* (note 14), 31, he recalls that ‘about 7 or 8 years since an able physician, namely Dr Puleston at Chester …went well to bed, and being suddenly taken in the night, was dead before I could come to him, though he lived in the same street’. Roger Puleston actually died not in 1661, as this might suggest, but on 8 January 1657 (Matthew H. Lee (ed.), *Diaries and Letters of Philip Henry* (London: Kegan Paul, 1882), 39, 41; burial register of St Peter’s Chester, 8 January 1657 ‘doctor in the art of phissicke’).

18.

*Ignota Febris*, *op. cit.* (note 4), (1698), 141. In 1690 he says he has ‘deserted the prescribing practice near thirty years’: *Test and Tryal of Medicines* (1690, see note 108), 6. Similarly in *Inquiries into the General Catalogue of Diseases* (1691, see note 93) he refers to ‘thirty years practice since [being a ‘prescriber to the shops’] in another thinking and working way, and proving of medicines’ (30).

19.
Wing M1518, ‘To the diseased’ (unpaginated). This is also appended to the 1668 edn of his *Treatise of Consumptions* (see n. 54).

20.
From a vast literature, see Alan G. Debus, ‘Chemists, Physicians, and Changing Perspectives on the Scientific Revolution’, *Isis*, 89 (1998), 66–81; Antonio Clericuzio, ‘From Van Helmont to Boyle’, *British Journal for the History of Science*, 16 (1993), 303–34; Michael Hunter, ‘Boyle Versus the Galenists’, *Medical History*, 41 (1997) 322–61; William R. Newman and Laurence M. Principe, *Alchemy Tried in the Fire* (Chicago, IL: Chicago University Press, 2005). I have followed Newman and Principe’s convention of using the spelling ‘chymical’ to distinguish this tradition from modern chemistry.10.1086/3839229588105

21.
W. Eamon, *Science and the Secrets of Nature* (Princeton, NJ: Princeton University Press, 1996); Vivian Nutton (ed.), *Medicine at the Courts of Europe, 1500–1837* (London: Routledge, 1990); Charles Webster, *Paracelsus* (New Haven, CT: Yale University Press, 2008).

22.
See Harold J. Cook, ‘Practical Medicine and the British Armed Forces after the “Glorious Revolution” ’, *Medical History*, 34 (1990), 1–26; Harold J. Cook, ‘Sir John Colbatch and Augustan Medicine’, *Annals of Science*, 47 (1990), 475–505; Harold J. Cook, *Matters of Exchange* (New Haven, CT, and London: Yale University Press, 2007); Patrick Wallis and David B. Haycock (eds), *Quackery and Commerce in Seventeenth-Century London*, *Medical History*, supplement 25, (2005); Mark Jenner and Patrick Wallis (eds), *Medicine and the Market in England and its Colonies *c*.1450–1850* (Basingstoke: Palgrave, 2007); Patrick Wallis, ‘Consumption, Retailing and Medicine in Early Modern London’, *Economic History Review*, 61 (2008), 26–53; Patrick Wallis, ‘Exotic Drugs and English Medicine’, *Social History of Medicine*, 25 (2012), 20–46.

23.
Patrick Wallis and T. Pirohakul, ‘Medical Revolutions? The Growth of Medicine in England, 1660–1800’, *Journal of Social History*, 49 (2016), 510–31, building on Ian Mortimer, *The Dying and the Doctors: The Medical Revolution in Seventeenth-Century England* (Woodbridge: Boydell and Brewer, 2009) and the forthcoming findings of the Wellcome-funded project ‘The Medical World of Early Modern England, Wales and Ireland *c*.1500–1715’.

24.
See above n. 1 and Harold J. Cook, ‘Good Advice and Little Medicine’, *Journal of British Studies*, 33 (1994), 1–33; Harold J. Cook, ‘Markets and Cultures’, *Transactions of the Royal Historical Society*, 6th ser., 21 (2011), 123–46.

25.
See Cook, *Decline*, *op. cit.* (note 1), 95–8; Frances Dawbarn, ‘Patronage and Power: The College of Physicians and the Jacobean Court’, *British Journal for the History of Science*, 31 (1998), 1–19; David L. Cowen, *Pharmacopoeias and Related Literature in Britain and America, 1618–1847* (Aldershot: Ashgate, 2001); H. Trevor-Roper, *Europe’s Physician* (New Haven, CT, and London: Yale University Press, 2006).10.1017/s000708749700316611619928

26.

Dmitri Levitin , ‘“Made up from Many Experimentall Notions”: The Society of Apothecaries, Medical Humanism and the Rhetoric of Experience in 1630s London’, Journal of the History of Medicine, 70 (2015), 549–587.10.1093/jhmas/jru02725281503

27.
The authoritative account of such conflicts pre-1640 is provided by Margaret Pelling, *Medical Conflicts in Early Modern London* (Oxford: Oxford University Press, 2003).

28.
A bibliography of the debate is given in Cook, ‘Physicians’, *op. cit.* (note 1), 169–71.

29.
Key works include: Charles Webster, ‘The College of Physicians: “Solomon’s House” in Commonwealth England’, *Bulletin of the History of Medicine*, 41 (1967), 393–412; Charles Webster, ‘English Medical Reformers of the Puritan Revolution’, *Ambix*, 14 (1967), 16–41; Charles Webster, *The Great Instauration: Science, Medicine, and Reform 1626–1660* (London: Duckworth, 1975); Charles Webster, *From Paracelsus to Newton* (Cambridge: Cambridge University Press, 1982); Harold J. Cook, ‘The Society of Chemical Physicians, the New Philosophy, and the Restoration Court’, *Bulletin of the History of Medicine*, 61 (1987), 61–77; Harold J. Cook, ‘The New Philosophy and medicine’, in David C. Lindberg and Robert S. Westman (eds), *Reappraisals of the Scientific Revolution* (Cambridge: Cambridge University Press, 1990), 397–436; Michael Hunter, *Establishing the New Science* (Woodbridge: Boydell and Brewer, 1989); Simon Shapin, *A Social History of Truth* (Chicago, IL: Chicago University Press, 1994).

30.

*Tutela Sanitatis sive Vita Protracta: The Protection of Long Life, and Detection of its Brevity* (1663) Wing M1516A, with second edn (1664) Wing M1517.

31.
Wing D857. The title also promises ‘*diagnostic signs to know the temperament and constitution of each body with a physical dyet and select counsels for each complexion, also indicating signs, advice and cautions for purging, vomiting, sweating and bleeding, with their proper effects and benefits aphoristically and methodically digested*’.

32.
It was traceable from the 1819 *Catalogue of the Library of the Medical and Chirurgical Society of London* (2nd part at 89) now incorporated in the Royal Society of Medicine Library at Tr. D. 77(5).

33.
Is it pure coincidence that seven of the nine letters of Degravere are an anagram of Everard?

34.
Extensive quotations from such publications can be found in C.J.S. Thompson, *The Quacks of Old London* (Philadelphia, PA: Lippincott, 1929), while they are analysed linguistically in Rebecca Mullini, *Healing Words: The Printed Handbills of Early Modern London Quacks* (Frankfurt am Main and New York: Lang, 2015).

35.

*Tutela Sanitatis*, *op. cit.* (note 30), introduction (unpaginated).

36.

*Ibid*.

37.

*Morbus Polyrhizos*, *op. cit.* (note 14), (1665), 93–4. Two separate publications, *Antiscorbutick Medecines. Exactly Prepared and Fitted for the Principal Cases that Occur in Practice* (1664) revised as *Antiscorbutick and Catholick Medicines* (London, 1666) are not in Wing but are included (with separate title pages) in the EEBO images of *Morbus* (1665) and *Morbus* (1666) respectively. John J. Keevil, *Medicine and the Navy*, Vol. 2 (Edinburgh: Livingstone, 1958), 119, regretted that this ‘sometime promising student at Cambridge’ but now ‘self-styled “Doctor of Hermetic Phylosophy and Physick”’ was not (Keevil claimed) really offering a serious treatise on scurvy at all but only advertising ‘the sale of his secret and useless “scorbute pill” ’. Only in two publications in 1666–7 (see nn. 48 and 54) did Maynwaring include ‘hermetical’ or ‘chymical philosophy’ in his title, otherwise always simply calling himself ‘doctor in physick’ or MD.

38.
Cook, *Decline*, *op. cit.* (note 1), 141–3.

39.

*Ibid.*, 167–8.

40.

*Medicus Absolutus Adespotos: The Compleat Physitian, Qualified and Dignified* (1668), Wing M1497, ‘To the Reader’ (unpaginated) and 86, 92.

41.

Edward Bolnest , *Aurora Chymica* (London, 1672), 147; Thomas Sherley, *A Philosophical Essay* (London, 1672), 141–2 (recommending *Praxis Medicorum Antiqua et Nova* (1671) as containing ‘the marrow of what hath been writ’ on the necessity of physicians ‘making and dispensing their own medicines …by Dr Cox, Dr Merrit, Dr Goderd [sic] and others, together with certain new and cogent arguments not formerly made use of’).

42.

*The History and Mystery of the Venereal Lues* (1673, see note 91), 214 (Bolnest); *Medicus Absolutus*, *op. cit.* (note 40), 135, 159–60 (Starkey) and 114 (Thomson); *Morbus Polyrhizos*, *op. cit.* (note 14) (1666), 34–5 (Thomson); *Praxis Medicorum* (see note 58), 20 (Thomson); George Thomson, *Loimologia* (London, 1665), 17; Thomson, *Plano-Pnigmos* (London, 1665), 10; Thomson, *Loimotomia* (London, 1666), 178–9; Thomson, *Misochymias Elenchos* (London, 1671), 41.

43.
‘I honour all that wear the gown in this faculty, and have a tender regard to their reputations, and wish they were all *medici absoluti* I meddle not with men but opinions and abuses in the practise of physick: I aim not to blast men but to reform errors’ (*Medicus Absolutus*, *op. cit.* (note 40), ‘To the reader’).

44.
BL Sloane MS 1786, ff. 116–28, quoted in Cook, *Decline*, *op. cit.* (note 1), 175, and Cook, ‘Physicians’, *op cit.* (note 1), 263.

45.

*Solamen Aegrorum, sive, Ternarius Medicamentorum Chymicorum ad Omnes fere Morbos Curandum* (1665) Wing M1513. This is his only work entirely in Latin, although he later published a short Latin tract summarising his preparations and his arguments for pharmaceutical medicine as *Pharmacopeia Domestica sive Repositorum Hippocraticum*, with a separate 1672 title page but continuously paginated (239–59) with the 1672 edn of *Morbus Polyrhizos*.

46.
Thomas O’Dowde, *The Poor Man’s Physician* (London, 1665), 94–7. An excellent summary of the extensive literature on this is provided by Cook, *Decline*, *op. cit* (note 1), 145–62, but see Peter Elmer, *Medicine and the Politics of Healing in Seventeenth-Century England* (Oxford: Oxford University Press, forthcoming).

47.

*An Advertisement from the Society of Chymical Physitians, touching Medicines by them Prepared, in Pursuance of His Majesties Command, for the Prevention, and for the Cure of the Plague* (London, 1665), reproduced in A.L. and D.C. Moote, *The Great Plague* (Baltimore, MD: Johns Hopkins University Press, 2006), 99.

48.

*Nova Medendi Ratio: A Short and Easie Method of Curing* (1666) Wing M1508A. He is styled ‘doctor in physick and chymical phylosophy’ on the title page.

49.
BL Add. MS 61649, ff. 182–183b. In *Test and Tryal, op. cit.* (note 18), (8) he claimed: ‘I have seen the highest Contagion that hath been known in *England* (Plague at *London,* 1665) and voluntarily ingaged therein from *first* to *last* when most Physicians ran away, and deserted the people in that Calamity: But I being provided with Antidotes preventive and Curative, and knowing it was my Duty, I therefore feared nothing; and visited those People, seized with the Pestilence (as I do now any other Disease) myself remaining in good health during the Contagion.’

50.
Anchitell Gray, *Debates of the House of Commons*, Vol. 1 (London, 1769), 403–5. The report states that the ‘sick house’ had cost £50, but was now an ‘alehouse’.

51.
It is not one of the five pesthouses discussed by Walter G. Bell, *The Great Plague in London* (London: Bodley Head, 1924), 38–9, 192–3 or Moote, *op. cit.* (note 47), 190–2.

52.
Alexander Taylor (ed.), *Works of Symon Patrick*, Vol. 9 (Oxford: Oxford University Press, 1858), 578 (summarised in Moote, *op. cit.* (note 47), 166).

53.
See Pelling, *op. cit.* (note 27), 245–74.

54.

*Tabidorum Narratio: a treatise of consumptions* (1667) Wing M1514 with 2nd edn (1668) Wing M1516. This described him as a ‘doctor in physick and hermetick phylosophy’.

55.
Joseph Foster (ed.), *London Marriage Licences 1521–1869* (London: Quaritch, 1887), 876. His wife Anne was the daughter of Elizabeth Ayloffe (née Peniston of Rochester, not far from Everard’s home town of Gravesend) of Foxton in Cambridgeshire, widow of James Ayloffe of Melbourn, Cambridgeshire (d. 1654). I owe this information to the kindness of Peter Elmer.

56.
He dedicated *The Method and Means of Enjoying Health* (1683) ‘from my study in the Inner Temple’ to the judges and the Inner Bench ‘having lately obtained the freedom of retirement within these walls (at vacant hours from medical employ) for fresh air and quiet recess’. Kenelm, son and heir apparent of Dr Everard Maynwairing of London, was admitted to the Inner Temple on 27 March 1683, but there is no other record of him.

57.

*Medicus Absolutus, op. cit.* (note 40).

58.

*Praxis Medicorum Antiqua & Nova: The Ancient and Modern Practice of Physick Examined, Stated, and Compared* (1671) Wing M1512.

59.

*Medicus Absolutus*, *op. cit.* (note 40), 39–50, 86.

60.

*Ibid.*, 169. His target is M[archamont] N[edham], *Medela Medicinae* (London, 1665), although he shared Nedham’s advocacy of chymical medicine.

61.

*Ibid.*, 64–9.

62.

*Vita Sana*, (see note 74) 101–7.

63.

*Medicus Absolutus*, *op. cit.* (note 40), 74, 103.

64.

*Praxis Medicorum*, *op. cit.* (note 58), 3, 9.

65.

*Ibid.*, 28 *et seq*; *Medicus Absolutus*, *op. cit.* (note 40), 51–63, 94.

66.

*Pharmacopoeian Physician’s Repository* (1669, see note 75), 82–5; *Test and Tryal*, *op. cit.* (note 18), 2–3.

67.

*Medicus Absolutus*, *op. cit.* (note 40), 63–77.

68.
Maynwaring was probably targeting George Castle, *The Chymical Galenist* (London, 1667).

69.

*Praxis Medicorum*, *op. cit.* (note 58), 24.

70.

*Ibid.*, 27–8; *Medicus Absolutus*, *op cit.* (note 40), 99–100, 109; *Useful Discoveries*, *op. cit.* (note 19), ‘To the Diseased’ (unpaginated).

71.

*Medicus Absolutus*, *op. cit.* (note 40), 85–92.

72.

*Ibid.*, 135, 159. For Starkey’s own dilemmas in establishing his professional identity and distinguishing himself from ‘empiricks’ such as Richard Mathews who marketed his products as their own, see William R. Newman, *Gehennical Fire* (Chicago, IL: Chicago University Press, 2003), 188–203.

73.
His books were not published by the leading publishers of works on chemistry, such as John Starkey and William Cooper, but by a range of London booksellers, most consistently Thomas Bassett (active 1659–93) of Fleet Street, best known for publishing law books (H.R. Plomer, *Dictionary of the Booksellers, Printers …1641–1667* (London: Bibliographical Society, 1907), 16).

74.

*Vita Sana & Longa: The Preservation of Health and Prolongation of Life Proposed and Proved* (1669) Wing M1519 with 2nd edn (1670) Wing M1520.

75.

*The Pharmacopoeian Physician’s Repository* (1669) Wing M1510 with 2nd edn (1670) Wing M1511.

76.
Two impressions, Wing M1498 and 1498A.

77.
Cook’s comments on Maynwaring reflect this. Initially he described his work as part of an attack on ‘learned medicine ... arguing for the importance of chemical physicians and of the new philosophy’ (*Decline*, *op. cit.* (note 1), 167), but, later, though noting that Maynwaring was one five ‘core’ people named as a member of the Society of Chymical Physicians on all three surviving lists, he cited his ‘Dublin medical degree’ and that he ‘wrote many books on medicine virtually all of which were sympathetic to the learned physicians and dietetic medicine, although he favoured some chemical medicines in therapy’ (Cook, ‘Society’, *op. cit.* (note 29), 71). More recently he has cited him as an example of someone who tried ‘incorporating the best of both worlds’, namely ‘dietetic medicine’ and ‘universalised therapeutics’ (‘Markets’, *op. cit.* (note 24), 136).

78.

*Medicus Absolutus*, *op. cit.* (note 40), 160; *Frequent, but Unsuspected Progress*, *op. cit.* (note 11), 166. However, *Ignota Febris*, *op. cit.* (note 4), (1698), rejects the ‘Archeus Helmontii’ as a separate subordinate to the ‘vital spirit’ (p. 14). Although Maynwaring’s medical philosophy was broadly Helmontian in character, he does not regularly cite Helmont or laud him as the key figure in modern medicine, as many chymical physicians did, and he writes in a plain style, eschewing the religious, mystical or alchemical language found in some of their works.

79.
Wing M1499.

80.

*Monarchia*, *op. cit.* (note 79), 24–8, 40–5, 49–55.

81.

*Frequent, but Unsuspected Progress*, *op. cit.* (note 11), 1–3, is even more explicit about the analogy with political order/rebellion.

82.

*Mystery*, (see note 111) 4–5.

83.
For his critique of Galenic purgatives, see *The Efficacy and Extent of True Purgation* (1696) Wing M1491.

84.

*Thesaurus Remediorum*, *op. cit.* (note 31), 24–7; *Tutela Sanitatis*, *op. cit.* (note 30), 77–120.

85.

*Morbus Polyrhizos*, *op. cit.* (note 14), (1665), 91–3. *Solamen Aegrorum*, *op. cit.* (note 45), also describes only 3 ‘medicamenta polychresta’ – scorbutic pills (p. 7), ‘elixir catholicon’ (p. 18) and ‘extractum sudorificum’ (p. 28). I have not seen his 8-page quarto pamphlet, *The practice of physick duly regulated. The extravagant invention, and destructive number of medicines, corrected and fitly reduced: for more safe, and successful conduct, in the methods of curing. By E.M. Med. D* (1690: ESTC R233837) as the only known copy is in the Folger Library and it is not available on EEBO.

86.

*Nova Medendi Ratio*, *op. cit.* (note 48), preface and 1, 7, 14, 24, 31–3.

87.
This analysis is expanded in *Test and Tryal*, *op. cit.* (note 18).

88.

*Medicus Absolutus*, *op. cit.* (note 40), 139.

89.

*Ibid.*, 150–1.

90.

*Ibid.*, 160–2; *Test and Tryal*, *op. cit.* (note 18) 4.

91.
Wing M1493 re-issued 1675. This was his only work to be published abroad (in Latin) as *Historia et Mysterium Luis Venerae* (Frankfurt am Main and Hamburg, 1675).

92.

*Frequent, but Unsuspected Progress*, *op. cit.* (note 11), 26.

93.
I*nquiries into the General Catalogue of Diseases Shewing the Errors and Contradictions of that Establishment with a New Scheme Representing more Truly, and Essentially, the Various Diseased State of Humane Nature* (1691) Wing M1496.

94.

*Frequent, but Unsuspected Progress*, *op. cit.* (note 11), 15–26 also argues this.

95.

*Inquiries*, *op. cit.* (note 93), 6–7.

96.
Wing M20B. Maynwaring’s authorship is confirmed by reference to ‘a late serious *tract,*intituled the *Ancient and Modern Practice of Physick, examined and compared,* &c. By the author of these medicines’.

97.
‘To the judicious and discreet reader’ (unpaginated).

98.
Cook, *Decline*, *op. cit* (note 1), 192–209, 233–5; Cook, *Trials of an Ordinary Doctor* (Baltimore, MD: Johns Hopkins University Press, 1994), 138–43.

99.
Wing M1490. He notes (p. 18), ‘I have not put my name to this narrative: (not that I am ashamed to own any thing here delivered) but for some reasons best known to my self: and desire to be excused herein …but if any person hath ought to object, against any thing here asserted, either as argument or matter of fact, let him produce; and the author hereof promises a reply and a further account of this grand remedy.’

100.
For example in *Mystery* (1694), see note 111, he says ‘the first essay of this comprehensive expedient was made upon my self (about ten years ago) and that in a desperate and extreme case’ (27).

101.

*Catholic Medicine*, *op. cit.* (note 99), 7.

102.

*Ibid.*, 8–10.

103.

*Ibid.*, 11.

104.

*Ibid.*, 15.

105.

*Ibid*.

106.

*Ibid.*, 17.

107.

*Serious Debate*, *op. cit.* (note 16), 8.

108.
(Wing M1515), 6–8.

109.

*Inquiries*, *op. cit.* (note 93), 8.

110.
See Cook references in notes 22 and 24.

111.
The subtitle continues: *Powerfully impregnated, and accuratly formed; to assist the regent principle, disabled, or impeded in vital government. Enabled hereby; more vigorously, and constantly to execute; the functions and daily operations; necessary to health, and life. For support of human bodyes, variously declining; and recovery out of a diseased state. The most useful, efficacious, and comprehensive expedient; (preventive and curative) against the painful diseased, and decays of human nature*. Wing M1506 with 2nd edn (1694) Wing M1507.

112.

*Ibid.*, 14–15.

113.

*Ignota Febris*,*op. cit.* (note 4), (1698), 146–7.

114.

*Flying Post*, 15 and 22 May 1701 and 18 and 27 September 1701; he had been at Denmark Court when *Ignota Febris*was advertised in the *Flying Post*, 4 November 1699.

